# Metabolic adaptations of immunosuppressive cells in cancer: mechanisms and therapeutic targets

**DOI:** 10.1038/s12276-026-01713-3

**Published:** 2026-05-07

**Authors:** Jihyoun Kim, Ji Min Shin, Yunju Um, Sujing Yuan, Seon Ah Lim

**Affiliations:** 1 Department of Life Science, Ewha Womans University, Seoul, Republic of Korea; 2 Institute of Modern Biology, Nanjing University, Nanjing, China; 3 Department of Thoracic Surgery, Nanjing Drum Tower Hospital, Medical School, Nanjing University, Nanjing, China; 4 Research Center for Cellular Homeostasis, Ewha Womans University, Seoul, Republic of Korea; 5 Multitasking Macrophage Research Center, Ewha Womans University, Seoul, Republic of Korea

**Keywords:** Tumour immunology, Immunotherapy

## Abstract

The tumor microenvironment harbors diverse immunosuppressive cell populations—including regulatory T cells, myeloid-derived suppressor cells, tumor-associated macrophages and other tolerogenic subsets—that drive immune evasion and therapeutic resistance. These cells are metabolically reprogrammed to sustain their suppressive function and survive under conditions of hypoxia, nutrient deprivation and oxidative stress. Importantly, their metabolic activity not only supports their own fitness but also creates a hostile environment that antagonizes effector T and natural killer cells by depleting essential nutrients, generating inhibitory metabolites, and altering signaling thresholds. This immunometabolic competition reinforces immune dysfunction and limits the efficacy of checkpoint blockade and adoptive cell therapies. Here we delineate the immunosuppressive cell types within the TME, their key metabolic adaptations and the mechanisms by which they suppress antitumor immunity. Finally, we discuss therapeutic strategies aimed at disrupting these metabolic programs to remodel the TME and enhance the success of current and next-generation immunotherapies. Collectively, understanding the metabolic crosstalk between suppressive and effector immune cells will provide new opportunities to design precision metabolic interventions and improve durable responses to cancer immunotherapy.

## Disrupting metabolic programs to boost antitumor immunity

The tumor microenvironment is a complex and suppressive niche that promotes tumor progression and resistance to therapy. This review explores how various immune cells within the tumor microenvironment, such as regulatory T cells, myeloid-derived suppressor cells, tumor-associated macrophages and tumor-associated neutrophils, adapt metabolically to maintain their suppressive functions. The authors detail the metabolic pathways these cells exploit, including fatty acid oxidation, glycolysis and amino acid catabolism, to thrive under nutrient-deprived and hypoxic conditions. Key findings reveal that these adaptations enable suppressive cells to outcompete effector T cells for nutrients, thereby impairing antitumor immunity. The study underscores the potential of targeting these metabolic pathways to enhance the efficacy of immunotherapies. Future directions include developing context-specific metabolic interventions and combining them with existing therapies to overcome resistance and improve patient outcomes.

This summary was initially drafted using artificial intelligence, then revised and fact-checked by the author.

## Introduction

The tumor microenvironment (TME) is a metabolically constrained and immunologically suppressive niche that drives tumor progression and therapeutic resistance^1,2^. It comprises malignant cells and diverse stromal and immune populations that interact through cytokine networks, extracellular vesicles and nutrient competition^3,4^. A key feature of the TME is the recruitment and functional reprogramming of suppressive immune subsets that disrupt immune surveillance^3^.

Among these, regulatory T (T_reg_) cells, myeloid-derived suppressor cells (MDSCs) and tumor-associated macrophages (TAMs) represent the major immunosuppressive populations. These cells not only accumulate in large numbers within tumors but are also functionally adapted to maintain suppressive activity under conditions of hypoxia, nutrient deprivation and oxidative stress—conditions that typically impair effector T cell and natural killer (NK) cell function^5,6^. By competing for nutrients, producing inhibitory metabolites and expressing immunosuppressive cytokines, these cells impair T cell receptor (TCR) signaling, limit costimulation and suppress antitumor cytokine production. Their enrichment in tumors has been consistently associated with poor prognosis, resistance to checkpoint blockade and failure of adoptive cell therapies^7,8^.

What distinguishes these suppressive cells is their ability to undergo profound metabolic rewiring. Unlike effector lymphocytes, whose functions are compromised by the metabolic constraints of the TME, suppressive cells exhibit remarkable plasticity in nutrient utilization, energy production and redox balance^9^. T_reg_ cells rely on fatty acid oxidation (FAO) and oxidative phosphorylation (OXPHOS) to sustain their suppressive phenotype in low-glucose environments^10^. MDSCs utilize arginine and cysteine depletion and reactive nitrogen species generation to inhibit effector T cells^11^. M2-like TAMs engage lipid metabolism to support their protumoral roles, including immunosuppression and angiogenesis^12^.

These metabolic adaptations are integrated with immunological and transcriptional programs via key regulators such as mTOR, AMPK, HIF-1α and SREBP. Tumor-derived signals, including lactate, transforming growth factor-β (TGF-β) and cytokines, further reinforce the suppressive and metabolically distinct identity of these cells. This creates a competitive and tolerogenic ecosystem that favors tumor survival over immune clearance^13,14^.

In this Review, we describe the major immunosuppressive cell types in the TME, characterize their metabolic adaptations, and highlight the mechanisms by which they impair effector function. We further explore emerging strategies to selectively target these metabolic programs to enhance the efficacy of current and next-generation immunotherapies. Understanding the metabolic basis of immune suppression in cancer offers critical insights for designing precision interventions that reshape the TME toward effective and durable antitumor immunity.

## The immunosuppressive landscape of the TME

### MDSCs

MDSCs are a heterogeneous population of immature myeloid cells that suppress immune responses in cancer and other pathological conditions^15^. They are broadly classified into monocytic MDSCs (M-MDSCs) and polymorphonuclear or granulocytic MDSCs (PMN-MDSCs). M-MDSCs resemble monocytes and have the capacity to differentiate into TAMs or dendritic cells (DCs), whereas PMN-MDSCs share phenotypic features with neutrophils and primarily exert suppressive functions in peripheral lymphoid organs.

Within the TME, M-MDSCs tend to be more prevalent than PMN-MDSCs, although the relative abundance of each subset varies depending on tumor type. M-MDSCs exert potent immunosuppressive effects by producing high levels of nitric oxide (NO), arginase1 (ARG1) and immunosuppressive cytokines. These mediators have a longer half-life compared with reactive oxygen species (ROS) and can act independently of direct cell–cell contact with T cells. Consequently, M-MDSCs are considered more broadly and robustly immunosuppressive than their PMN counterparts^16^.

**Fig. 1:**
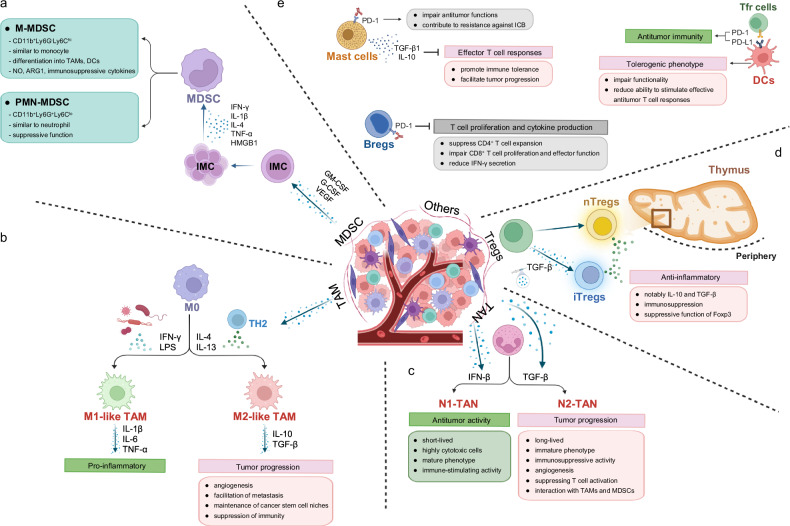
The immunosuppressive landscape of the TME. **a** In the TME, immature myeloid cells (IMCs) expand in response to primary signals such as GM-CSF, G-CSF and VEGF. MDSCs are subsequently activated by secondary signals, including inflammatory cytokines and DAMPs. MDSCs are categorized into two subsets—M-MDSCs and PMN-MDSCs—which exert distinct functions within the TME. **b** TAMs differentiate into either M1-like or M2-like phenotypes. M1-like TAMs are induced by LPS and IFN-γ and secrete cytokines such as TNF-α, IL-1β and IL-6, promoting pro-inflammatory responses. By contrast, M2-like TAMs are induced by cytokines such as IL-4 and IL-13, which are secreted by Th2 cells, and they adopt an immunosuppressive phenotype. **c** TANs are classified into N1 and N2 phenotypes. IFN-β promotes polarization toward N1 TANs, which exhibit antitumor properties. By contrast, TGF-β promotes polarization toward N2 TANs, which display protumoral, immunosuppressive phenotypes. **d** T_reg_ cells are divided into nT_reg_ cells and iT_reg_ cells. nT_reg_ cells develop in the thymus, whereas iT_reg_ cells arise in peripheral tissues in response to exogenous TGF-β. Both subsets secrete IL-10 and TGF-β, which contribute to immunosuppression and support the suppressive function of Foxp3⁺ T_reg_ cells. **e** Mast cells impair antitumor functions and contribute to resistance against ICB by expressing PD-1. They release TGF-β1 and IL-10, which inhibit effector T cell responses. B_reg_ cells can suppress T cell proliferation and cytokine production under PD-1/PD-L1 signaling. PD-L1^+^ B_reg_ cells can suppress CD4^+^ T cell expansion and impair CD8^+^ T cell proliferation. They can also reduce IFN-γ secretion. Meanwhile, DCs interact with follicular T_reg_ cells by binding to each other via PD-1/PD-L1. They also exhibit tolerogenic phenotypes in the tumor-cell-associated environment. In association with tumor cells, they impair functionality and reduce the ability to stimulate an effective antitumor T cell response.

Phenotypically, PMN-MDSC is defined as CD11b^+^Ly6G^+^Ly6C^lo^, and M-MDSC is defined as CD11b^+^Ly6G^-^Ly6C^hi^ (ref. ^17^). The accumulation and activation of MDSCs within tumors require two distinct groups of signals. The first group signal promotes the expansion of immature myeloid cells and is driven by chronic inflammation and tumor- or bone marrow-derived factors such as GM-CSF, G-CSF, M-CSF, SCF, vascular endothelial growth factor (VEGF) and polyunsaturated fatty acids. These signals converge on transcriptional regulators including STAT3, STAT5, IRF8, C/EBPβ, Notch, adenosine receptor A2b, NLRP3 and RB1. The second signal drives pathological activation through inflammatory cytokines and danger-associated molecular patterns (DAMPs; for example, interferon-γ (IFN-γ), interleukin (IL)-1β, IL-6, tumor necrosis factor-α (TNF-α) and high-mobility group box 1 (HMGB1)), primarily engaging NF-κB and STAT pathways to stabilize suppressive function^18^ (Fig. 1a). Together, these mechanisms enable MDSCs to exert potent immunosuppressive effects across diverse tumor types, thereby contributing to immune evasion and tumor progression.

### TAMs

Macrophages are among the most abundant immune cell populations within the TME^19^. TAMs arise from two main developmental origins: monocyte-derived TAMs, which originate from bone marrow hematopoietic stem cells and circulate as peripheral monocytes before infiltrating tumors, and embryonically-derived, tissue-resident TAMs, which differentiate from yolk sac progenitors during development^20^. While tissue-resident TAMs often exhibit pro-inflammatory characteristics, monocyte-derived TAMs tend to adopt immunosuppressive phenotypes in tumors^20^. Recruitment of circulating monocytes into the TME is driven by tumor-derived chemotactic signals. Key factors such as colony-stimulating factor 1 (CSF1) and vascular endothelial growth factor A (VEGFA) promote the infiltration and differentiation of monocyte precursors, thereby contributing to tumorigenesis. The CCL2–CCR2 signaling axis plays a particularly important role in the mobilization and recruitment of monocytes from the bloodstream to tumor sites^20^.

Macrophages exhibit remarkable plasticity and adapt dynamically to local environmental cues. In response to distinct cytokine milieus, they polarize toward either classically activated (M1) or alternatively activated (M2) states^21^. Thus, macrophage polarization states are conceptually distinct from developmental origin, as both monocyte-derived and embryonically derived macrophages can adopt M1-like or M2-like phenotypes depending on contextual signals within the TME^22^. TAMs, however, rarely conform to this binary model; instead, they exist along a continuum of activation states ranging from pro-inflammatory M1-like to immunosuppressive M2-like phenotypes. Within the TME, cytokines such as IL-4 and IL-13, secreted by T helper 2 (Th2) cells, drive polarization toward an M2-like state. IL-4 signaling activates STAT6 through the JAK–STAT6 pathway, reinforcing M2 polarization and consolidating the immunosuppressive TAM phenotype^23,24^. These M2-polarized TAMs secrete anti-inflammatory mediators such as IL-10 and TGF-β, thereby fostering tumor progression through angiogenesis, metastasis, maintenance of cancer stem cell niches, and suppression of antitumor immunity^25^.

By contrast, microbial products such as lipopolysaccharide (LPS) and cytokines such as IFN-γ induce M1 polarization and pro-inflammatory activity, marked by production of cytokines such as TNF-α, IL-1β and IL-6 (Fig. 1b). Through such context-dependent phenotypic plasticity, TAMs emerge as central regulators of immune suppression and tumor progression in the TME.

### TANs

Tumor-associated neutrophils (TANs) represent an emerging immune cell population with context-dependent roles in tumor biology^26^. Functionally, TANs are broadly classified into two phenotypes: N1 TANs, which exert antitumor effects, and N2 TANs, which promote tumor progression^26^. N1 TANs are short-lived, highly cytotoxic and characterized by a mature phenotype with strong immune-stimulatory capacity. By contrast, N2 TANs exhibit prolonged survival, retain an immature phenotype and display immunosuppressive, pro-angiogenic and prometastatic properties.

The polarization of TANs is primarily shaped by cues from the TME. TGF-β promotes differentiation toward the N2, protumoral phenotype, thereby reinforcing immune suppression and supporting tumor growth. Conversely, IFN-β has been shown to drive polarization toward the N1, antitumor state, while simultaneously inhibiting acquisition of immunosuppressive traits^27,28^.

Within tumors, N2-polarized TANs contribute to immune evasion through multiple mechanisms. They suppress T cell activation and proliferation by releasing immunosuppressive cytokines and chemokines, enhancing T_reg_ cell recruitment and attenuating antigen presentation^28^. Moreover, crosstalk between N2 TANs and other suppressive immune populations, including TAMs and MDSCs, amplifies the establishment of an immunosuppressive milieu^29^ (Fig. 1c). Collectively, these findings underscore the remarkable plasticity of TANs and their capacity to reinforce immune evasion within the TME.

### T_reg_ cells

T_reg_ cells are a specialized subset of CD4⁺ T lymphocytes that play a pivotal role in maintaining immune tolerance and preventing excessive immune activation in various contexts, including within the TME^30^. T_reg_ cells are broadly categorized into two major subsets: naturally occurring T_reg_ cells (nT_reg_ cells), which develop in the thymus, and inducible T_reg_ cells (iT_reg_ cells), which arise in the periphery under the influence of factors such as TGF-β^31,32^. Both nT_reg_ and iT_reg_ cells contribute to immunosuppression primarily through the secretion of anti-inflammatory cytokines, most notably IL-10 and TGF-β^33^.

The transcription factor *Foxp3* is indispensable for T_reg_ development, lineage stability and suppressive function. The stable expression of *Foxp3* serves as a key marker for T_reg_ cells, most notably in the CD4⁺CD25⁺ population. Ectopic expression of *Foxp3* is both necessary and sufficient to induce a T_reg_ phenotype in naive T cells, highlighting its central and nonredundant role in establishing T_reg_ lineage identity^34^. Recent studies have revealed that the transcriptional regulation of *Foxp3* is governed by several conserved noncoding sequences (CNSs) within the *Foxp3* gene locus—specifically CNS0, CNS1, CNS2 and CNS3^35,36^. CNS0 promotes IL-2-dependent induction of *Foxp3* expression via the IL-2–STAT5 axis during early differentiation stages. CNS1 is dispensable for thymic nT_reg_ development but is essential for peripheral iT_reg_ induction, often acting in concert with CNS3. CNS2 contributes to the long-term stability of *Foxp3* expression in mature iT_reg_ cells, while CNS3 acts as an early enhancer to initiate *Foxp3* transcription during lineage commitment.

Together, these regulatory elements orchestrate a tightly controlled transcriptional program that sustains T_reg_ development and function (Fig. 1d). This molecular network is crucial for maintaining immune homeostasis and becomes particularly relevant in the TME, where T_reg_ cells accumulate and reinforce tumor-induced immune evasion^37^.

### Other suppressive immune subsets in the TME

Beyond T_reg_ cells, MDSCs, TAMs and TANs, several additional immune cell subsets within the TME contribute to immune evasion and therapeutic resistance. Among these, tumor-associated mast cells, regulatory B (B_reg_) cells and tumor-associated DCs have emerged as key regulators of immunosuppression^38^.

Mast cells, traditionally known for their roles in allergy and tissue remodeling, are increasingly recognized for their immunomodulatory activities in cancer. Within tumors, they express programmed cell death protein 1 (PD-1), which impairs their antitumor functions and contributes to resistance against immune checkpoint blockade (ICB)^39^. They also release immunosuppressive cytokines such as IL-10 and TGF-β1, further promoting immune tolerance and facilitating tumor progression by dampening effector T cell responses^40^.

B_reg_ cells represent a distinct B cell subset that exerts suppressive effects primarily through the inhibition of T cell proliferation and cytokine production. B_reg_ cells have been shown to suppress CD4⁺ T cell expansion and IFN-γ secretion in tumor-bearing hosts^41^. One of the key mechanisms underlying their immunosuppressive function involves PD-1/PD-L1 signaling. In invasive breast cancer models, PD-L1⁺ B_reg_ cells impair CD8⁺ T cell proliferation and effector function, highlighting their potential as a therapeutic target^42^. Moreover, PD-L1⁺ B_reg_ cells exhibit enhanced capacity to modulate cytokine production compared with conventional B_reg_ cells, further reducing IFN-γ production by CD4⁺ and CD8⁺ T cells^43^.

DCs, although classically regarded as potent antigen-presenting cells, can acquire tolerogenic phenotypes within the TME. In cancers such as bladder cancer, they often display impaired antigen presentation and fail to prime effective T cell responses^44^. In addition, PD-L1 expression on these cells suppress the differentiation and function of follicular regulatory T (T_fr_) cells, thereby modulating humoral immunity^45^. Importantly, blockade of PD-L1 signaling has been shown to restore T cell priming and represents a promising therapeutic approach^46^ (Fig. 1e).

Collectively, these additional immunosuppressive populations highlight the cellular diversity of immune regulation within tumors. Understanding their specific contributions to immune escape and therapy resistance will be critical for developing more comprehensive and effective immunotherapeutic strategies.

## Metabolic programs of immunosuppressive cells in TME

### MDSCs

**Fig. 2:**
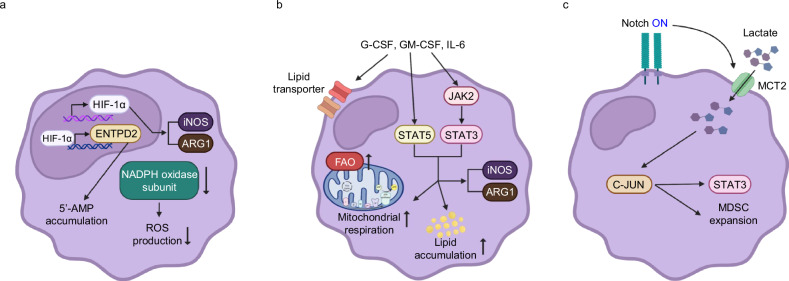
Metabolic adaptations of MDSCs in TME. **a** Under hypoxic conditions, HIF-1α is stabilized, leading to increased expression of iNOS and ARG1. Hypoxia also suppresses NADPH oxidase activity, resulting in reduced ROS production. In addition, HIF-1α induces ENTPD2 expression, leading to the accumulation of extracellular 5′-AMP. **b** Tumor-derived cytokines, including G-CSF, GM-CSF and IL-6, upregulate lipid transporter expression and enhance FAO. These signals activate the STAT5 and JAK2/STAT3 pathways, which further promote mitochondrial respiration and increase lipid accumulation in MDSCs. These pathways also enhance iNOS and ARG1 expression. **c** Lactate accumulation in the TME promotes MDSC activation and expansion through a Notch–MCT2–c-Jun–STAT3 signaling axis.

Hypoxia, a hallmark of the TME, plays a critical role in augmenting the immunosuppressive activity of MDSCs. Under hypoxic conditions, tumor-infiltrating MDSCs exhibit stronger suppression of both antigen-specific and nonspecific T cell responses compared with their splenic counterparts, as observed in models such as CC10 transgenic lung cancer^47^. Conditioned medium from hypoxic hepatocellular carcinoma cells enhances MDSC migration, highlighting hypoxia’s role in their recruitment^48^. Mechanistically, hypoxia alters ROS metabolism and upregulates immunosuppressive enzymes including ARG1 and inducible NO synthase (iNOS). Notably, reduced expression of NADPH oxidase subunits (gp91^phox^ and p47^phox^) under hypoxia limits ROS production. Hypoxia-inducible factor-1α (HIF-1α) is central to this process, its genetic ablation in MDSCs enhances antitumor immunity in mice^47^. In addition, HIF-1α regulates extracellular nucleotide metabolism by inducing ENTPD2, which promotes accumulation of 5′-AMP, thereby facilitating MDSC recruitment and tumor growth in human hepatocellular carcinoma samples^49^ (Fig. 2a). HIF-2α also contributes by upregulating CCL26, which attracts CCR1⁺ MDSCs to tumors^48^. Collectively, hypoxia promotes MDSC accumulation and suppressive function via metabolic and transcriptional reprogramming.

MDSCs in the TME undergo lipid metabolic reprogramming to sustain their immunosuppressive function. These cells show upregulation of lipid transporters (for example, CD36, LRP1 and VLDLR) and fatty acid metabolism genes, driven by tumor-derived cytokines such as G-CSF, GM-CSF and IL-6^50^. Functionally, tumor-infiltrating MDSCs demonstrate reduced glucose uptake (as measured by 2-NBDG (2-(N-(7-nitrobenz-2-oxa-1,3-diazol-4-yl)amino)-2-deoxyglucose) staining) but increased fatty acid uptake, indicates a preferential reliance on FAO over glycolysis^51^. Mechanistically, stimulation of bone marrow cells with G-CSF, GM-CSF and IL-6 activates STAT5 and JAK2–STAT3 signaling, promoting lipid accumulation and mitochondrial respiration. Pharmacological inhibition of these pathways reduces lipid uptake, impairs ARG1and iNOS activity, and attenuates MDSC-mediated suppression in human^50^ (Fig. 2b). Similarly, blockade of carnitine palmitoyltransferase 1 (CPT1) with etomoxir diminishes fatty acid uptake and ATP production, thereby limiting the immunosuppressive activity of MDSCs in mouse model^51^. Together, these findings establish lipid metabolic reprogramming as a central regulator of MDSC function in tumors.

Lactate accumulation is a defining metabolic hallmark of the TME, largely driven by the Warburg effect. Elevated lactate levels not only reflect altered tumor metabolism but also promote MDSC recruitment and activation. Instead of altering the NAD⁺/NADH ratio through pyruvate conversion, excess lactate activates the Notch–MCT2–c-Jun signaling pathway, thereby enhancing STAT3 activity and MDSC differentiation^52,53^. MDSCs lacking RBP-J, a transcriptional mediator of Notch signaling, show impaired suppressive capacity toward T cells^52^. Clinical studies further link the Notch–MCT2–c-Jun axis with MDSC expansion and lung adenocarcinoma progression^52^ (Fig. 2c). In addition to modulating T cell responses, lactate-enriched conditions enhance the ability of MDSCs to inhibit NK cell cytotoxicity and prevent DC maturation in both human and mouse model^54^. Thus, lactate serves not merely as a byproduct of tumor metabolism but as a potent immunoregulatory metabolite that amplifies MDSC differentiation and function.

### TAMs

**Fig. 3:**
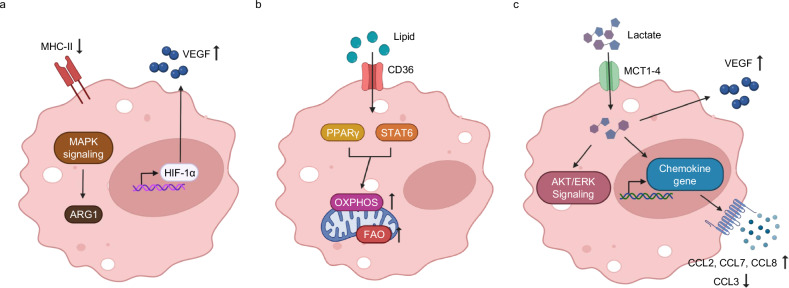
Metabolic adaptations of TAMs in TME. **a** Hypoxia promotes ARG1 expression through MAPK-dependent signaling pathways. In parallel, hypoxic conditions stabilize HIF-1α, leading to increased expression of VEGF. Reduced expression of MHC class II molecules under hypoxia further contributes to immune evasion. **b** Lipid uptake mediated by CD36 activates PPARγ and STAT6 signaling, thereby promoting OXPHOS and FAO, **c** Lactate uptake via monocarboxylate transporters (MCT1–4) enhances VEGF production and activates AKT/ERK signaling pathways. Lactate-driven chemokine modulation increases the expression of CCL2, CCL7 and CCL8, while suppressing CCL3 production.

Hypoxia is a key factor influencing the polarization and functional heterogeneity of TAMs^21^. In particular, oxygen gradients within tumors drive phenotypic divergence of TAMs. In mouse model, macrophages residing in hypoxic regions express elevated levels of ARG1, a hallmark of the M2-like, immunosuppressive phenotype. Gene set enrichment analyses from RNA sequencing datasets reveal that ARG1 expression in TAMs is induced via activation of the MAPK signaling pathway^55^. In addition, VEGF is strongly upregulated in TAMs under hypoxia, primarily mediated by HIF-1α-dependent transcriptional activation^56^. Murine TAM subsets also differ based on MHC class II expression. MHC-II^low^ TAMs, enriched in hypoxic niches, can process antigen but fail to efficiently prime naïve T cells, thereby contributing to immune evasion. Conversely, MHC-II^hi^ TAMs rely more heavily on iNOS-mediated pathways for their functional roles, including immunomodulation and promotion of angiogenesis^21^. Together, these observations highlight that hypoxia-driven TAM heterogeneity plays a central role in shaping an immunosuppressive TME (Fig. 3a).

To adapt to the nutrient-deprived conditions of the TME, TAMs undergo metabolic reprogramming, notably through enhanced lipid uptake and utilization. Lipids serve as alternative energy sources, taken up via scavenger receptors such as CD36. Genetic ablation of *CD36* in TAMs results in reduced support of tumor cell proliferation, implicating this transporter in protumoral metabolic crosstalk^57^. Key transcriptional regulators such as STAT6 and PPARγ orchestrate the metabolic phenotype of M2-like TAMs by promoting FAO and mitochondrial OXPHOS^58^ (Fig. 3b). In addition, Hedgehog signaling has been shown to regulate FAO in TAMs. Inhibition of Hedgehog signaling downregulates PPARγ and PGC1β, impairs FAO and reduces ATP production, thereby limiting immunosuppressive function. This metabolic shift is accompanied by mitochondrial dysfunction and diminished mitochondrial membrane potential^59^. These findings collectively underscore the importance of lipid metabolism and mitochondrial fitness in maintaining TAM-mediated immunosuppression.

Lactate, a major metabolic byproduct in tumors, plays a crucial role in polarizing TAMs toward an immunosuppressive phenotype. Interestingly, HIF-1α can be stabilized not only under hypoxic conditions but also under normoxic conditions in the presence of lactate, leading to M2 polarization^60^. TAMs cultured with conditioned media from Lewis lung carcinoma or B16 melanoma cells upregulate lactate transporter expression (MCT1–4) and exhibit increased VEGF production^56^. Moreover, lactate triggers activation of the AKT and ERK signaling pathways in a concentration-dependent manner. In vivo, co-injection of lactate and macrophages into a subcutaneous tumor model significantly enhanced tumor growth^61^. TAMs stimulated by lactate also produce higher levels of CCL2, CCL7 and particularly CCL8, while CCL3 levels decrease (Fig. 3c). Notably, CCL8 binds to CCR5 on tumor cells, promoting tumor growth and metastasis^61^. These results indicate that tumor-derived lactate reprograms TAMs through AKT–ERK activation and chemokine modulation, thereby facilitating tumor progression and immune escape.

### TANs

**Fig. 4:**
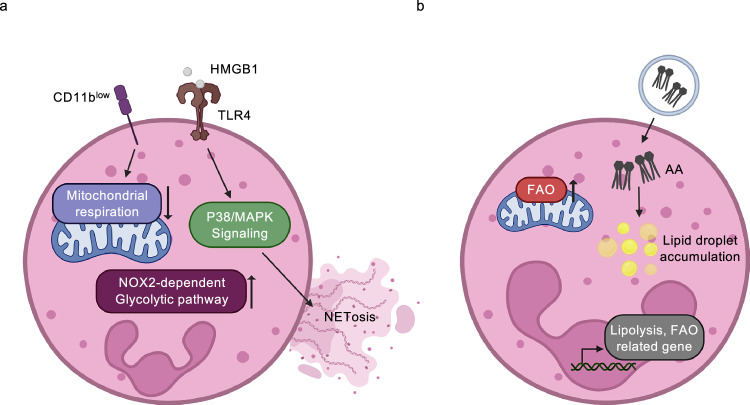
Metabolic adaptations of TANs in TME. **a** Under hypoxic conditions, HMGB1 engages TLR4, activating p38/MAPK signaling and promoting NET formation. In parallel, hypoxia-sensitive CD11b^low^ TANs undergo a metabolic shift from mitochondrial respiration to a NOX2-dependent glycolytic pathway. **b** Within the TME, TANs upregulate genes involved in lipolysis and FAO and acquire the capacity to uptake tumor-derived lipids. Extracellular-vesicle-mediated delivery of arachidonic acid contributes to lipid droplet accumulation, providing metabolic substrates that sustain TAN activation.

Hypoxia in the TME influences the phenotype and function of TANs. One of the hallmark responses of TANs to hypoxic stress is the induction of neutrophil extracellular traps (NETs), which have been increasingly implicated in tumor progression and metastasis in patients. Clinical data indicate that elevated NET formation is associated with poor prognosis across multiple cancer types^62^. In gastric cancer, exposure to hypoxia-conditioned media leads to a marked increase in neutrophil infiltration and NET release^63^. Mechanistically, this process is mediated by the Toll-like receptor 4 (TLR4)/p38 MAPK signaling pathway activated in response to HMGB1. Under hypoxic conditions, HMGB1 translocates to the cytoplasm of tumor cells and acts as a DAMP that engages TLR4 on neutrophils, triggering p38 MAPK activation and promoting NETosis^64^. In addition to NET induction, hypoxia also reprograms the metabolic profile of TANs. In mouse models, CD11b^low^ TANs, a subset particularly sensitive to hypoxic cues, shift their energy metabolism from mitochondrial respiration to a NOX2-dependent glycolytic pathway^65^ (Fig. 4a). Moreover, in murine breast cancer models, TANs overexpressing aconitate decarboxylase 1 (ACOD1) contribute to tumor growth and metastasis by suppressing ferroptosis in tumor cells^66^. Collectively, these findings illustrate the metabolic and functional plasticity of TANs under hypoxia and highlight their contribution to immune evasion and tumor progression.

Lipid metabolism plays a crucial role in neutrophil development and function and is profoundly altered in the TME. In particular, TANs are capable of transferring lipids to cancer cells, thereby supporting their metabolic needs during metastasis. Lung mesenchymal cells have been shown to induce lipid droplet accumulation in TANs during the pre-metastatic phase. Upon interaction with lung mesenchymal cells, TANs upregulate lipolysis- and FAO-related genes, including *Lipe*, *Atgl*, *Lipa* and *Cpt1b*, facilitating enhanced energy production and supporting metastatic colonization in breast cancer models^67^. A distinct population of resistant TANs (RTANs) have also been identified in triple-negative breast cancer following therapy. RTANs exhibit elevated uptake of tumor-derived arachidonic acid, delivered via extracellular vesicles, resulting in lipid accumulation and enhanced immunosuppressive function, as observed in both mouse models and human patients (Fig. 4b). Transcriptomic and pathway analyses, including ingenuity pathway analysis, reveal that RTANs suppress key pathways related to T cell activation and cytotoxicity, underscoring their role in dampening antitumor immunity^68^. These findings suggest that, in lipid-rich environments, TANs undergo metabolic reprogramming that reinforces their immunosuppressive phenotype and contributes to therapeutic resistance.

### T_reg_ cells

**Fig. 5:**
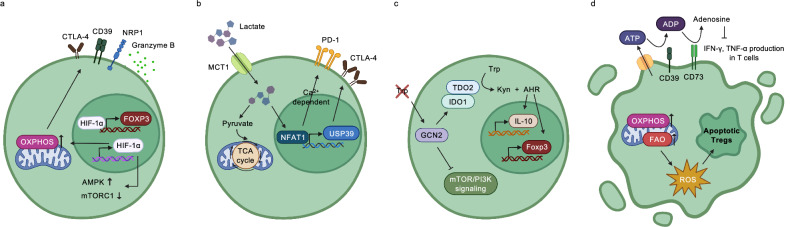
Metabolic adaptations of T_reg_ cells in TME. **a** HIF-1α binds to the *Foxp3* promoter to enhance its transcription. It also promotes OXPHOS and AMPK activation while suppressing mTORC1 signaling, thereby supporting the expression of suppressive molecules such as Granzyme B, CD39, CTLA-4 and NRP1. **b** Lactate uptake via MCT1 converts into pyruvate and enters TCA cycle. Lactate also promotes NFAT1 nuclear translocation, which induces USP39-mediated RNA splicing essential for CTLA-4. In addition, PD-1 expression is also upregulated by the Ca^2+^-dependent NFAT1 signaling axis. **c** Tryptophan depletion activates GCN2, which inhibits PI3K/mTOR signaling and induces the expression of IDO1 and TDO2, enzymes that convert tryptophan into kynurenine. Kynurenine activates AHR, thereby upregulating *Foxp3* and *IL-10*. **d** Enhanced OXPHOS and FAO promote ROS generation, and increased ROS induce T_reg_ apoptosis. Apoptotic T_reg_ cells release ATP and converted to adenosine via CD39 and CD73. Adenosine suppresses IFN-γ and TNF-α production by effector T cells.

Hypoxia profoundly influences the differentiation, stability and function of T_reg_ cells. The *Foxp3* gene, the master regulator of T_reg_ identity, contains multiple hypoxia response elements, suggesting transcriptional regulation by hypoxia-inducible factors (HIFs). Among these, HIF-1α is highly expressed in naive CD4⁺ T cells and remains stable upon T_reg_ differentiation, while HIF-2α expression declines. Under hypoxic conditions, HIF-1α is stabilized by the inhibition of prolyl hydroxylase domain (PHD) enzymes^69^. Stabilized HIF-1α can bind directly to the *Foxp3* promoter, promoting *Foxp3* transcription and enhancing T_reg_ differentiation and suppressive function^70^. In glioblastoma models, HIF-1α activation correlates with increased T_reg_ accumulation and tumor progression^71^. However, conflicting evidence indicates that HIF-1α may repress *FOXP3* while favoring Th17 differentiation^72^. HIF-1α also regulates T_reg_ metabolism by promoting glycolysis to support migration and maintaining lipid metabolism to sustain immunosuppressive activity. HIF-1α-deficient T_reg_ cells exhibit reduced migration, impaired OXPHOS and diminished tumor control^73^. These metabolic changes are accompanied by AMPK activation and mTORC1 suppression, and inhibition of fatty acid metabolism results in decreased expression of suppressive markers such as Granzyme B, CD39, CTLA-4 and neuropilin-1 (NRP1)^74^ (Fig. 5a). Furthermore, HIF-2α has also been implicated in T_reg_ function. In MC38 colon adenocarcinoma models, HIF-2α-deficient T_reg_ cells exhibit impaired suppressive capacity^75^. Together, these findings indicate that both HIF-1α and HIF-2α are key regulators of T_reg_ metabolism and function under hypoxic conditions.

Lactate accumulation in the TME, reshapes T_reg_ metabolism and enhances their suppressive function. Tumor-infiltrating T_reg_ cells utilize extracellular lactate via monocarboxylate transporter 1 (MCT1), which is highly expressed on their surface^76^. Once internalized, lactate is converted to pyruvate and enters the tricarboxylic acid (TCA) cycle or is used for phosphoenolpyruvate synthesis, fueling gluconeogenic pathways. Lactate availability also promotes the expression of immune checkpoint receptors, thereby enhancing T_reg_-mediated immunosuppression^77^. Lactate uptake via MCT1 leads to nuclear translocation of NFAT1 and upregulation of RNA-splicing components such as ubiquitin-specific peptidase 39 (USP39), facilitating splicing-mediated expression of CTLA-4. In colorectal cancer patients, high levels of USP39-driven CTLA-4 expression are observed in T_reg_ cells^78^. Similarly, in acute myeloid leukemia, lactate induces T_reg_ cell accumulation and upregulates PD-1 expression through a Ca²⁺-dependent NFAT1 signaling axis^79^ (Fig. 5b). MCT1 deletion in T_reg_ cells results in reduced tumor growth and improved responsiveness to immunotherapy, underscoring lactate’s role as an alternative energy source that reinforces immunosuppressive function^77^. Taken together, the differential metabolic reliance of T_reg_ cells—those with high lactate avidity exhibit enhanced suppressive activity, whereas those dependent on glucose display diminished function. Conversely, lactate can also exert context-dependent immunostimulatory effects by sustaining stem-like CD8⁺ T cells. Acting as a histone deacetylase (HDAC) inhibitor, lactate enhances H3K27 acetylation at the *Tcf7* super-enhancer locus, thereby maintaining TCF-1 expression and supporting durable antitumor immunity. In addition, lactate has recently been identified as a signaling metabolite that regulates gene expression through histone lactylation, linking metabolic states to immune cell fate decisions^80^.

Amino acid availability in the TME plays a crucial role in shaping T_reg_ function. While tumors consume large amounts of amino acids to support rapid proliferation, they also modulate the local amino acid milieu by releasing certain metabolites^81^. One of the most well-characterized mechanisms involves tryptophan (Trp) depletion. Trp scarcity activates the general control nonderepressible 2 (GCN2) kinase pathway, which inhibits the mTOR–PI3K axis and induces expression of indoleamine 2,3-dioxygenase 1 (IDO1) and tryptophan 2,3-dioxygenase (TDO2)^82^. These enzymes convert Trp into kynurenine (Kyn), a ligand for the aryl hydrocarbon receptor (AHR). AHR activation promotes *Foxp3* and *IL-10* expression, reinforcing the immunosuppressive function of T_reg_ cells^83^ (Fig. 5c). In glioblastoma, tumor cells release excessive glutamate through the xCT (SLC7A11) antiporter, which enhances T_reg_ activation by increasing expression of CD69, CD154 and Ki-67^84^. These observations highlight that amino acid metabolism, particularly involving Trp and glutamate, modulates T_reg_ activity and supports immune evasion.

T_reg_ cells adapt to the harsh TME—characterized by low glucose and high lactate—by shifting their metabolism toward OXPHOS and FAO, processes that generate ROS^85^. ROS accumulation, tightly regulated by NRF2-dependent antioxidant responses, influences T_reg_ survival and can trigger apoptosis. Apoptotic T_reg_ cells release ATP, which is subsequently converted to adenosine through the CD39/CD73 ectonucleotidase axis. Adenosine signaling suppresses effector T cell cytokine production, including IFN-γ and TNF-α, thereby amplifying immunosuppression^86^ (Fig. 5d). Thus, oxidative stress not only limits T_reg_ survival but paradoxically enhances immunosuppression through the release of apoptotic byproducts.

## Metabolic antagonism between suppressive and effector cells

### Nutrient competition

Nutrients such as glucose and amino acids are essential for both suppressive and effector immune cells to support their development and function. Within the TME, not only tumor cells but also immunosuppressive populations such as T_reg_ cells and TAMs consume glucose, exacerbating nutrient depletion. Glycolysis is critical for T cell activation, survival and effector function, and glucose scarcity directly impairs these processes. Indeed, T cells exposed to glucose-deprived TME exhibit reduced IFN-γ production, a lower glycolysis, and decreased uptake of glucose analog 2-NBDG, indicating suppressed glycolytic activity^87,88^. In parallel, amino acid depletion contributes to immune suppression. MDSCs express high levels of ARG1, which hydrolyzes arginine into ornithine and urea, depleting arginine required for T cell proliferation^89^. In addition, MDSCs frequently upregulate indoleamine 2,3-dioxygenase (IDO), which catabolizes tryptophan into kynurenine. This pathway mediates immunosuppression via two mechanisms: tryptophan depletion activates the stress sensor GCN2, halting T cell proliferation, while kynurenine promotes T_reg_ differentiation and suppresses effector T cell responses through activation of the AHR^90^. These nutrient-depleting strategies not only impair effector T cell proliferation but also reinforce the suppressive cell compartment, amplifying immune evasion within the TME. Importantly, recent therapeutic strategies aim to convert such metabolic competition into a therapeutic opportunity rather than broadly suppressing shared metabolic pathways. Unlike tumor cells, which often exhibit rigid metabolic dependencies such as glutamine addiction, effector T cells retain substantial metabolic plasticity and can reprogram their bioenergetic pathways under nutrient stress. For example, the glutamine antagonist prodrug JHU-083 selectively starves metabolically inflexible tumor cells while preserving, or even enhancing, antitumor T cell function. Under glutamine-restricted conditions, effector T cells adapt by engaging compensatory oxidative metabolic programs and preferentially acquire a highly activated, long-lived memory phenotype, thereby sustaining antitumor immunity and improving therapeutic efficacy^91^. Collectively, these findings illustrate how metabolic competition within the TME can be leveraged to create a therapeutic window in which tumor metabolism is selectively constrained while effector T cell function is maintained or enhanced.

### Metabolite-mediated suppression

The TME is enriched with immunosuppressive metabolites such as lactate, ROS and adenosine, which are produced by both tumor cells and suppressive immune populations. These metabolites disrupt the function of effector immune cells, including T and NK cells. Due to the Warburg effect, lactate accumulates in the TME and enhances the suppressive activity of T_reg_ cells, TAMs, TANs and MDSCs, while impairing effector function. T and NK cells exposed to high lactate levels exhibit reduced secretion of IFN-γ and granzyme B^92^. In T cells, lactate is transported via MCT1, lowering intracellular pH and disrupting metabolic homeostasis. Once internalized, lactate is converted into pyruvate by lactate dehydrogenase (LDH), a reaction that reduces NAD⁺ to NADH, increasing the NADH/NAD⁺ ratio. This shift impairs glycolysis by limiting GAPDH activity, ultimately inhibiting T cell proliferation^93^. Adenosine also suppresses immune responses in the TME. Apoptotic cells release ATP, which is sequentially converted into adenosine by the ectonucleotidases CD39 and CD73, highly expressed on T_reg_ cells and MDSCs. Adenosine then binds to the A2A receptor (A2AR) on T cells, elevating intracellular cAMP levels. This suppresses TCR signaling, proliferation and cytokine production, resulting in T cell anergy or functional paralysis^94,95^. Collectively, these immunosuppressive metabolites reshape the TME into a metabolically hostile niche that favors suppressive cells while silencing effector responses.

### Interference with effector metabolic reprogramming

Effector immune cells dynamically reprogram their metabolism in response to environmental cues and activation signals. In a resting state, most immune cells rely on OXPHOS for energy. Upon activation, however, they shift to aerobic glycolysis to rapidly generate ATP and supply biosynthetic precursors for proliferation^96^. In T cells, this metabolic reprogramming is driven by TCR stimulation through CD3/CD28, leading to increased glucose uptake and glycolytic flux via activation of the PI3K–AKT–mTOR pathway^97,98^. Suppressive immune cells interfere with this reprogramming through multiple mechanisms. T_reg_ cells express CTLA-4, which competes with CD28 for binding to CD80/CD86, thereby preventing PI3K-AKT activation^99^. In addition, PD-L1 on MDSCs engages PD-1 on T cells, further suppressing this glycolytic switch^8,100^. Arginine depletion by ARG1-expressing MDSCs and TANs also inhibits mTOR activation, impairing the metabolic transition required for T cell proliferation and effector function^101^. Thus, suppressive immune cells not only consume critical nutrients but also actively block the metabolic rewiring necessary for effector activation, creating a layered barrier to antitumor immunity.

## Therapeutic targeting of immunosuppressive cell metabolism

### Targeting MDSC metabolism

Recent lipidomic analysis has shown that MDSCs accumulate specific lipid species that contribute to their immunosuppressive effects on T cells. In preclinical models, targeting fatty acid transport protein 2 (FATP2) with the FATP2 inhibitor lipofermata in MDSCs reduces lipid accumulation and decreases ROS production in MDSCs, thereby attenuating their suppressive activity and limiting tumor growth.

Moreover, inhibition of FATP2 has been reported to enhance the efficacy of anti-PD-L1 therapy in murine models, accompanied by increased CD107a expression and reduced PD-L1 levels on tumor-infiltrating CD8⁺ T cells^102^. ARG1 is another key metabolic enzyme implicated in myeloid cell-mediated immune suppression. Pharmacological inhibition of arginase using INCB001158 (also known as CB-1158) has been shown in experimental models to modulate the immune microenvironment and enhance antitumor immune responses, particularly in combination with ICB^103^. However, recent clinical studies demonstrated that, despite clear pharmacodynamic target engagement—including restoration of systemic arginine levels—antitumor efficacy of INCB001158 remained limited, underscoring the redundancy and complexity of arginine metabolism within the TME^104^. These findings highlight the challenges of translating arginine-targeting strategies into durable clinical benefit. In addition, mTOR-driven glycolysis is crucial to support suppressive function of tumor-infiltrating M-MDSCs in mice. Targeting glycolysis with rapamycin treatment attenuates the suppressive activity of tumor-associated M-MDSCs and effectively impedes tumor growth^105^. Collectively, these findings suggest that targeting MDSC metabolism can modulate immunosuppressive programs; however, successful therapeutic translation will probably require rational combination strategies and biomarker-guided patient stratification rather than metabolic monotherapy alone.

### Targeting TAM metabolism

Given that metabolic alterations are the primary drivers of macrophage suppression in the TME, repolarizing TAMs through metabolic reprogramming offers a promising opportunity to activate tumoricidal immunity. Targeting the TAM lipid metabolic pathway through etomoxir treatment may suppress tumor growth by promoting the generation of M1 macrophages^57^. The knockdown of *SLC3A2* in lung adenocarcinoma cells decreased arachidonic acid levels in the TME, thereby hindering the M2 polarization of macrophages^106^. Glutamine synthetase (GS) is a key enzyme that promotes M2-like macrophage differentiation by increasing intracellular glutamine levels. Inhibition of GS using methionine sulfoximine (MSO) has been shown to shift IL-10-treated macrophages from an M2-like to an M1-like phenotype^107^. Pharmacological inhibition or genetic deletion of *Gpr132*, the sensor of lactate, could attenuate M2-like phenotype in TAMs and impair the tumor formation of breast cancer cells^108,109^. 2-Deoxy-d-glucose (2-DG), an inhibitor of the glycolytic pathway, decreases anti-inflammatory M2 macrophage polarization and prevents disease progression in murine models^109^. CD40 signaling activation by monoclonal antibody rewires metabolic circuits to enhance the antitumorigenic polarization of TAMs and boost the antitumor response^110^. Although most strategies for targeting TAMs remain in the preclinical stage, several therapeutic approaches-such as CD40 agonists, HDAC inhibitors and PI3Kγ inhibitors are currently being evaluated in clinical trials in combination with immune checkpoint therapy^111^. The evidence overwhelmingly supports that metabolic reprogramming is a viable and powerful strategy to counteract macrophage-mediated immunosuppression. The future of immunotherapy will undoubtedly involve these combination strategies that target both the effector and suppressor components of the immune system.

### Targeting TAN metabolism

Selective inhibition of fatty acid metabolism by FATP2 or ferroptosis inhibition abrogated the immunosuppressive function of TANs and reduced tumor growth. When combined with ICB, this strategy not only counteracts the immunosuppressive activity of TANs but also more effectively delays tumor progression and enhances the antitumor efficacy of anti-PD-1 therapy^112,113^. AMPK, a key regulator of energy metabolism, plays a critical role in neutrophil glycolysis^114^. Studies have shown that targeting the AMPK signaling pathway induces metabolic reprogramming in TANs and reduces their immunosuppressive activity. In mouse models of colorectal cancer, activation of the AMPK pathway by retinoic acid (RA) suppressed the glycolytic capacity of TANs and delayed tumor progression^115^. Targeting these specific metabolic pathways offers a highly rational and effective approach to disrupt this alliance, reverse immunosuppression and unlock the full potential of existing immunotherapies. The future of cancer treatment lies in these sophisticated combination strategies that simultaneously target multiple cell types within the TME.

### Targeting T_reg_ metabolism

The inhibition of glycolysis by various pharmacological agents such as 2-DG or galloflavin has been reported to decrease the function and proliferation of T_reg_ cells, enhances immune responses to cancer^116,117^. Targeting the kynurenine pathway through inhibition of IDO1 has been shown in preclinical models to modulate T_reg_-mediated immune suppression^118,119^. However, clinical studies using the IDO1 inhibitor epacadostat, including the phase III ECHO-301/KEYNOTE-252 trial, demonstrated no improvement in progression-free or overall survival with epacadostat plus pembrolizumab compared with pembrolizumab alone in patients with melanoma, indicating that IDO1 inhibition alone does not consistently translate into improved antitumor efficacy^120^. Alterations in fatty acid metabolism, including fatty acid synthesis and FAO, have been proposed as additional strategies to disrupt T_reg_ metabolic fitness. In experimental models, inhibition of fatty acid synthesis using 5-tetradecyloxy-2-furoic acid or blockade of FAO using etomoxir has been shown to reduce T_reg_ suppressive activity^121^. However, interpretation of etomoxir-based studies requires caution, as etomoxir exerts CPT1a-independent off-target effects on mitochondrial metabolism, particularly at high concentrations^122^. Moreover, genetic ablation of *Cpt1a* does not fully recapitulate the phenotypes observed with pharmacological inhibition, underscoring important differences between genetic and pharmacological approaches when interpreting the role of FAO in T_reg_ biology^123^. In addition, targeting lipid uptake pathways such as CD36 impairs fatty acid metabolism in T_reg_ cells and enhances antitumor immune responses in murine tumor models, with emerging evidence also reported in human systems^124^.

Collectively, these findings across immunosuppressive cell populations highlight the central role of metabolic pathways in shaping immune suppression within the TME. However, accumulating preclinical and clinical evidence indicates that effective therapeutic translation will likely require context-specific targeting, improved metabolic specificity and rational combination strategies rather than broad metabolic inhibition alone (Table 1).

**Table 1:** Strategies for targeting immunosuppressive cell metabolism for cancer therapy.

Metabolic vulnerability	Target	Drug(s)/targeting method(s)	Indication(s)	Notes	Clinical status	Reference
Fatty acids	FATP2	Lipofermata	MDSCs TANs	Decreased Lipid/ROS accumulation; reduced tumor growth; synergized with ICB	Preclinical	^112,113,125^
	CPT1	Etomoxir	TAMs T_reg_ cells	Inhibited lipid metabolism (FAO); M1-like TAM reprogramming and reduced T_reg_ suppressive function; high-dose off-target effects	Preclinical	^57,121,122^
	SLC3A2	CRISPR/Cas9	TAMs	Inhibited M2 macrophage polarization	Preclinical	^106^
Glucose	mTOR	Rapamycin	MDSCs	Reduced tumor growth	Phase I/II NCT01195922	^105^
	Glycolysis	2-DG	TAMs Tregs	Inhibited M2 macrophage polarization; decreased T_reg_ function and proliferation	Phase I/II NCT00633087	^109,116,117^
	Glycolysis	Galloflavin	T_reg_ cells	Decreased T_reg_ function and proliferation;	Preclinical	^117^
Amino acid	ARG1	CB-1158, INCB001158	MDSCs	Enhanced antitumor immune responses; synergized with ICB	Phase I NCT02903914	^104,126^
	GS	MSO	TAMs	Promoted M1 macrophage polarization	Preclinical	^107^
Lactate	LDHA	Oxamic acid	TAMs	Reduced lactate production; attenuated M2 macrophage polarization; prevented disease progression	Preclinical	^108^
Energy metabolism	AMPK	RA	TANs	Reduced tumor growth	Phase II NCT02403778	^115^
Kynurenine	IDO1	Epacadostat	T_reg_ cells	Preclinical studies showed inhibition of T_reg_-mediated immunosuppression; no clinical benefit was observed in phase III trials when combined with anti-PD-1 therapy	Phase III NCT02752074	^118–120^

## Conclusion and perspective

Immunosuppressive cells within the TME—including T_reg_ cells, MDSCs, TAMs and TANs—exhibit remarkable metabolic plasticity that enables them to thrive under nutrient-deprived, hypoxic and acidic conditions. By contrast, effector immune cells such as CD8⁺ T cells and NK cells are metabolically disadvantaged in this hostile environment, leading to impaired function and reduced antitumor efficacy. This metabolic antagonism is a critical barrier to the success of current immunotherapies.

Recent studies have highlighted how these suppressive populations exploit key metabolic pathways—such as FAO, glycolysis, amino acid catabolism and lactate utilization—to maintain their immunosuppressive phenotypes. Moreover, the TME is shaped by metabolic byproducts such as lactate, ROS and adenosine, which further inhibit effector cell function and reprogramming. These insights have opened new avenues for metabolic intervention to reprogram the immune landscape of tumors.

Moving forward, several critical areas warrant further investigation. First, there is an urgent need to identify context-specific metabolic checkpoints that selectively impair suppressive cells without compromising effector cell function. Second, the development of metabolic imaging tools and spatial metabolomics will be essential to decipher the dynamic interplay between immune cell subsets in vivo. Third, combinatorial strategies integrating metabolic modulators with immune checkpoint inhibitors or adoptive cell therapies should be systematically evaluated to overcome resistance mechanisms and enhance durable responses. Fourth, some compounds may have off-target effects, for example, although inhibition of long-chain FAO with etomoxir has been widely used to dissect the metabolic role of FAO in lymphocytes, Raud, O’Connor and colleagues demonstrated—using Cpt1a genetic ablation models—that the effects of etomoxir on T cell differentiation and function are independent of Cpt1a expression^122,123^. This finding highlights the potential for off-target effects of etomoxir on cellular metabolism, particularly when used at high concentrations. Finally, translating these findings into clinically actionable biomarkers and therapies will require a multidisciplinary approach encompassing immunology, oncology, systems biology and bioengineering.

Ultimately, targeting the unique metabolic vulnerabilities of immunosuppressive cells represents a promising strategy to tip the immunological balance in favor of antitumor immunity and improve patient outcomes across a broad spectrum of cancers.
